# Acute Phase Proteins in Dogs with Natural Infection by *Trypanosoma cruzi*

**DOI:** 10.3390/tropicalmed8060299

**Published:** 2023-05-31

**Authors:** Pilar Rivadeneira-Barreiro, Roberto Montes-de-Oca-Jiménez, Pablo Zambrano-Rodríguez, Juan Carlos Vázquez-Chagoyán, Adriana del Carmen Gutiérrez-Castillo, Luis Pardo-Marin, Lorena Franco-Martínez, José Joaquín Cerón, Silvia Martínez-Subiela

**Affiliations:** 1 Centro de Investigación y Estudios Avanzados en Salud Animal, Facultad de Medicina Veterinaria y Zootecnia, Universidad Autónoma del Estado de México, Km 15.5 Carretera Panamericana Toluca-Atlacomulco, Toluca 50200, Mexico; 2 Departamento de Veterinaria, Facultad de Ciencias Veterinarias, Universidad Técnica de Manabí, Portoviejo 130105, Ecuador; 3 Interdisciplinary Laboratory of Clinical Analysis, Interlab-UMU, Regional Campus of International Excellence ‘Campus Mare Nostrum’, University of Murcia, 30100 Murcia, Spain

**Keywords:** acute phase proteins, Chagas disease, dogs, ferritin, PON-1, *Trypanosoma cruzi*

## Abstract

Acute phase proteins have been used as tools for the diagnosis, monitoring, and prognosis of several diseases in domestic animals. However, the dynamics of these proteins in infection by *Trypanosoma cruzi*, the causative agent of Chagas disease in dogs, is still unknown. The aim of this study was to determine concentrations of acute phase proteins (C-reactive protein, haptoglobin, ferritin and paraoxonase-1) in dogs in a coastal town of Ecuador, with natural *Trypanosoma cruzi* infection with or without seroreactivity of *Ehrlichia canis*, *Ehrlichia ewingii*, *Anaplasma phagocytophilum*, *Anaplasma platys*, *Borrelia burgdorferi* and *Dirofilaria immitis*. For the detection of *Trypanosoma cruzi* serum antibodies, two different antigen-based enzyme-linked immunosorbent assay tests were implemented. For the detection of seroreactivity of *Ehrlichia canis*, *Ehrlichia ewingii*, *Anaplasma phagocytophilum*, *Anaplasma platys*, *Borrelia burgdorferi* and *Dirofilaria immitis*, an IDEXX SNAP^®^ 4Dx^®^ test was used. To determine the concentration of C-reactive protein and ferritin, an immunoturbidimetric assay was used; haptoglobin concentration was measured using a commercial colorimetric method validated in dogs; a spectrophotometric method was used to determine the serum concentration of paraoxonase-1. Results showed a reduction in the serum levels of paraoxonase-1 in *Trypanosoma cruzi*-seroreactive dogs, either with or without seroreactivity to other vector-borne diseases. A serum ferritin increment was observed in *Trypanosoma cruzi*-seroreactive dogs with seroreactivity to any other vector-borne diseases. Our findings suggest that paraoxonase-1 levels are reduced in *Trypanosoma cruzi*-seroreactive dogs without evident clinical signs of Chagas disease, despite their seroreactivity to the other vector-borne diseases studied. These findings could indicate an oxidative stress response in *Trypanosoma cruzi*-seroreactive dogs with no evident signs of inflammation.

## 1. Introduction

Chagas disease, caused by the protozoan *Trypanosoma cruzi* (*T. cruzi*), a hemoflagellate protozoan that belongs to the phylum Sarcomastigophora, subphylum Mastigophora, Class Zoomastigophorea, order Kinetoplastida, and the Trypanosomatidae family is a zoonotic disease transmitted by triatomines, insects from the order Hemiptera, that belong to the Reduviidae family [1]. These insects are endemic in a region that includes most Latin American countries, and puts approximately 6 to 7 million people at risk of contracting the infection [2,3]. The initial diagnosis of Chagas disease, also referred to as American trypanosomiasis, occurred in 1909 in Lassance, Minas Gerais state, Brazil [4].

Infection was initially confined to America, from the southern states of the United States of America down to Argentina, but then it spread to some countries of other continents due to human migration, where it was transmitted via non-vectorial means such as blood transfusion, tissues transfer, congenitally, or via laboratory accidents [5]. It can naturally affect more than 150 mammals, including humans and dogs, the latter being the main reservoir in the domestic cycle [6]. Dogs are the most appropriate experimental model to study Chagas disease because they develop *T. cruzi* infection in a similar way to humans [7,8]. In both humans and dogs, clinical manifestations often involve lymphadenopathy, splenomegaly, acute myocarditis, and chronic cardiomegaly [8]. The diagnosis is normally conducted using parasitological and/or serological methods [9].

Ehrlichiosis, anaplasmosis, heartworm disease, and Lyme disease are other vector-borne diseases (VBDs) that affect dogs, caused by *Ehrlichia canis* (*E. canis*), *Ehrlichia ewingii* (*E. ewingii*), *Anaplasma phagocytophilum* (*A. phagocytophilum*), *Anaplasma platys* (*A. platys*) and *Dirofilaria immitis* (*D. immitis*), respectively, and characterized also by being zoonotic. The vectors of these diseases are mosquitoes (*Culex* spp., *Aedes* spp., and *Anopheles* spp.) or ticks (*Rhipicephalus sanguineus*) that can transmit these pathogens to different species of mammals, including humans and dogs [10]. These diseases have some common symptoms, such as pale mucous membranes, fever, lethargy, anorexia, cardiac disorders, hemorrhages, lymphadenomegaly, splenomegaly, and nervous signs [11,12,13]. These VBDs are often diagnosed through microscopy, serological tests, and polymerase chain reaction (PCR), among other means of diagnosing these associated VBDs [10].

The acute phase response (APR) is a function of the innate immune system that occurs as an immediate response to an alteration in homeostasis, caused by inflammations, infections, neoplastic processes, immunological disorders, trauma, and stress [14,15]. It represents the first line of defense of the organism and involves the formation of proinflammatory cytokines, which stimulate hepatocytes to produce acute phase proteins (APPs). In the APR there is an increase in the production of certain APPs such as C-reactive protein (CRP), haptoglobin (Hp), serum amyloid A, ferritin (positive APPs), and a decrease in other APPs such as albumin and paraoxonase-1 (PON1) (negative APPs) [16,17,18,19].

APPs have been recognized as potential diagnostic markers for several decades. Rudolf Virchow, a German physician, introduced the fifth cardinal signal of inflammation in the mid-19th century [20]. However, it was not until the mid-20th century that the measurement of APPs became widespread with the introduction of automated protein analysis techniques. However, some evidence suggests limitations in the use of APPs for diagnosis purposes. For example, APPs are not specific to a particular disease, as they can be elevated in response to a variety of stimuli [21], and thus it may require additional testing to confirm the underlying cause. Additionally, the levels of APPs may vary depending on the timing of the sample collection, as their levels can peak at different times during the acute phase response [22].

APPs have been studied in canine infectious diseases such as leishmaniasis [23,24], dirofilariosis [25,26,27,28], parvovirosis [29], ehrlichiosis [30], leptospirosis [31], canine babesiosis [32], and African trypanosomiasis [33]. In leishmaniosis, an increase in the concentration of CRP, Hp, and ferritin was observed [24,34]; in heartworm disease there was an increase in CRP and a decrease in PON-1 [27]; and, in canine monocytic ehrlichiosis, there were variations in the concentrations of CRP, Hp, ferritin, and PON-1 [35].

Although APP studies have been conducted in dogs with experimental infection with *Trypanosoma brucei* [33], to the best of our knowledge there are no studies of these proteins in dogs with natural infection with *T. cruzi* with seroreactivity to ehrlichiosis, anaplasmosis, and dirofilariasis, since these diseases can coexist in the study area. However, quantification of APPs could provide a helpful tool for the diagnosis of Chagas disease when trying to determine life prognosis or monitoring disease evolution and/or recovery after treatment, as reported for other different pathological processes [36,37]. Therefore, the main objective of this study was to determine concentrations of different APPs in naturally *T. cruzi*-infected dogs with or without seroreactivity to other VBDs.

## 2. Materials and Methods

### 2.1. Study Area

Collection of samples was carried out in Colon and Calderon, two urban communities of Portoviejo city in the province of Manabi, Ecuador, during 2018. Manabí is located on the pacific coast of Ecuador, and it is considered not only an endemic zone for Chagas disease in humans [38], but also endemic for ehrlichiosis, anaplasmosis and heartworm disease, since this location has the climatic conditions for the proliferation of triatomines, mosquitoes, and ticks [39,40,41].

Sampling was conducted with convenience, where the only inclusion criteria was to have dog owners with informed consent for animals’ blood sampling and analysis. Originally, the study considered only the research of dogs seroreactivity to *T. cruzi*. However, after suspecting the presence of concomitant VBDs in several of these animals, the aim of this study was reconfigured to study the interactions in APPs serological levels in animals with concomitant VBDs seroreactivity.

### 2.2. Sampling Process

Samples from 85 dogs were included in the study (30 samples from dogs located in Colon and 55 samples from dogs in Calderon) to detect seroreactivity against *T. cruzi*, *E. canis*, *E. ewingii*, *A. phagocytophilum*, *A. platys*, *B. burgdorferi*, and *D. immitis*. The 30 dogs from Colon were from a previous study that attempted to detect *T. cruzi* and other VBDs [42]. All hemolized, lipemic, and/or icteric samples were discarded and not included in the study. The 85 dogs in this study were randomly chosen for examination without any criteria of preference. Inclusion criteria included dogs from any sex, aged from 3 months to 10 years, disregarding breed information.

### 2.3. Blood Samples Collection

The blood samples (5 mL) were obtained through jugular vein puncture (with a 20-gauge needle) and were collected in Vacutainer tubes without anticoagulant. Serum was obtained through centrifugation at 1500× *g* for 10 min, then it was stored and frozen at −80 °C until assayed.

### 2.4. Anti-T. cruzi Antibody

The best way to diagnose Chagas disease is to directly detect *T. cruzi* from the blood by using parasitological methods. However, sometimes the parasitemias are so low that this method becomes impractical. Therefore, when parasitological methods are not an option, the World Health Organization (WHO) recommends the use of at least two serologic tests based on different immunologic principles to confirm diagnosis [9,43]. Therefore, in the present study, to detect anti-*T. cruzi* antibodies, two enzyme-linked immunosorbent assay (ELISA) tests based on different immunologic designs were used (Accutrack, Laboratorios Lemos S.R.L. Buenos Aires, Argentina), ‘Chagas recombinant microELISA’ (based on Recombinant protein used as plate-sensitizing antigens) and the ‘Chagas microELISA test system’ (based on Trypomastigote lysates used as plate-sensitizing antigens), with modifications according to Aparicio, et al. [44] as follows: To prepare the samples, they were mixed with a dilution buffer containing a PBS solution, 0.05% Tween-20, and 3% nonfat milk, and were diluted to 1/100. Next, 200 μL of each diluted sample was added in duplicate to 96-well ELISA plates and incubated at 37 °C for 30 min followed by 20 min at room temperature. After this, the wells were washed six times with a washing buffer provided by the manufacturer. Then, a secondary antibody called horseradish peroxidase-conjugated sheep anti-dog (IgG) was added to the wells. This antibody was diluted 1:5000 in the dilution buffer. The plates were then incubated at 37 °C for 30 min followed by 30 min at room temperature. The reaction was developed by adding a TMB and hydrogen peroxide solution premixed at a 1:1 ratio, following the manufacturer’s instructions. The samples were then incubated in the dark for 20 min at room temperature. To stop the reaction, 100 µL of sulfuric acid (1M) was added to each well. The color change was recorded at 450 nm using an Epoch microplate reader equipped with Gene 5 v.2.0 software. The cutoff value was determined as the average reading of negative samples plus 0.1 optical density. The same procedure was used for both ELISA tests.

In order to consider if *T. cruzi* infected a patient, it must have reacted positive to two positive serologic tests [9,43]. In the present study, to be considered a *T. cruzi*-seroreactive animal, the animal had to react positive to both ELISA test kits used. A total of 45 animals resulted serologically positive to *T. cruzi* following this criteria.

### 2.5. Seroreactivity against Etiological Agents of Four Vector-Borne Diseases

A commercial kit (IDEXX SNAP^®^ 4Dx^®^ Plus) was used for the detection of serum antibodies against *E. canis*, *E. ewingii*, *A. phagocytophilum*, *A. platys*, and *B. burgdorferi*, as well as antigens from *D. immitis.* Samples were processed according to manufacturer’s instructions as follows: Before starting the test, the sample temperature must be between 18–25 °C. In a new sample tube dispense three drops of the sample using a pipette, then add four drops of conjugate to the sample tube and mix the content by inverting it three to five times. Place the SNAP device on a flat surface and add the content of the sample tube to the sample well. The sample will move across the result window and should reach the activation circle in 30–60 s. Once the color appears in the activation circle, it is necessary to press the activator until it is flushed with the device body.

Two groups of samples were formed: Group 1, which included samples from *T. cruzi*-seronegative (*T. cruzi* ^N^) dogs (*n* = 40 dogs); and Group 2, which included *T. cruzi*-seroreactive (*T. cruzi* ^P^) dogs (*n* = 45 dogs). The second group was subdivided into two subgroups: subgroup 2a, (*n* = 23 dogs) without seroreactivity to other diseases VBDs (VBDs ^N^), and group 2b (*n* = 22 dogs), with seroreactivity to one or more other VBDs (VBDs ^P^).

### 2.6. Evaluation of Acute Phase Proteins

CRP concentration was determined using a human immunoturbidimetric test (CRP OSR6147 Olympus Life and Material Science Europe GmbH, Hamburg, Germany) previously standardized for dogs [45]. Haptoglobin was evaluated using a hemoglobin-binding method (Tridelta Phase range haptoglobin kit; Tridelta Development Limited, Ireland). Ferritin concentrations were estimated using an immunoturbidimetric assay with polyclonal anti-human ferritin antibodies (Tina-quant Ferritin, Roche Diagnostic, Mannheim, Gemany) validated for use in dogs [24]. PON-1 activity was analyzed following a previously described spectrophotometric assay [46]. Determinations of APPs were performed using an automated biochemistry analyzer (Olympus AU600; Olympus Diagnostica GmbH, Hamburg, Germany).

### 2.7. Statistical Analysis

For clinical samples the D’Agostino and Pearson omnibus normality test was performed to evaluate the normality of distribution in terms of whether collected samples fit in a parametric or nonparametric distribution. Then, data were log-transformed, and an unpaired Student *t* test was used to compare acute phase protein concentrations between *T. cruzi*-seroreactive vs. seronegative dogs and to compare *T. cruzi*-seroreactive dogs with and without seroreactivity to other VBDs. An ordinary one-way ANOVA followed by an uncorrected Fisher LSD test were performed to compare acute phase protein values obtained in negative vs. seroreactive dogs with seroreactivity to others VBDs and seroreactive dogs without seroreactivity to others VBDs. A *p*-value < 0.05 was statistically significant in all cases. Statistical analyses were performed using GraphPad Prism 8 software (GraphPad Software Inc., San Diego, CA, USA).

## 3. Results

### 3.1. Trypanosoma cruzi-Seroreactive Dogs with Seroreactivity to Others Vector-Borne Diseases

**Figure 1:**
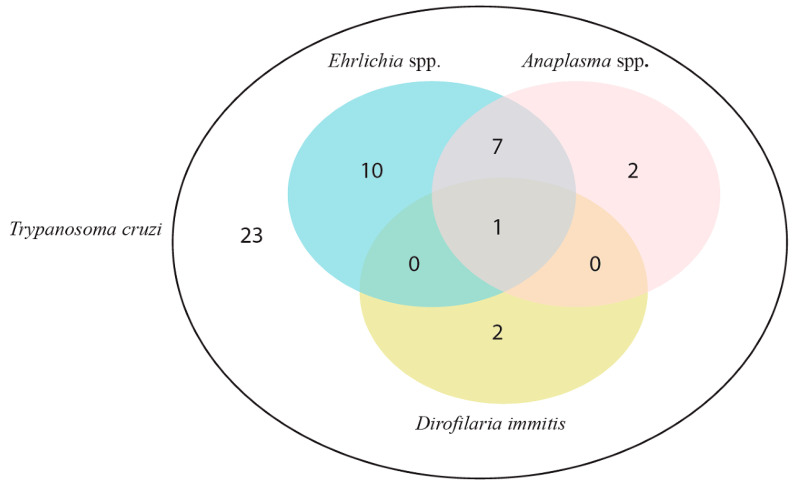
*T. cruzi*-seroreactive dogs with seroreactivity to *Ehrlichia* spp. *Anaplasma* spp. and *Dirofilaria immitis*.

The results of the serologic diagnostic are shown in Figure 1.

### 3.2. PON-1 Concentrations in T. cruzi-Seronegative Dogs (Group 1) vs. T. cruzi-Seroreactive Dogs (Group 2) and T. cruzi-Seroreactive Dogs without (Group 2a) and with Seroreactivity to Other Vector-Borne Diseases (Group 2b)

**Figure 2:**
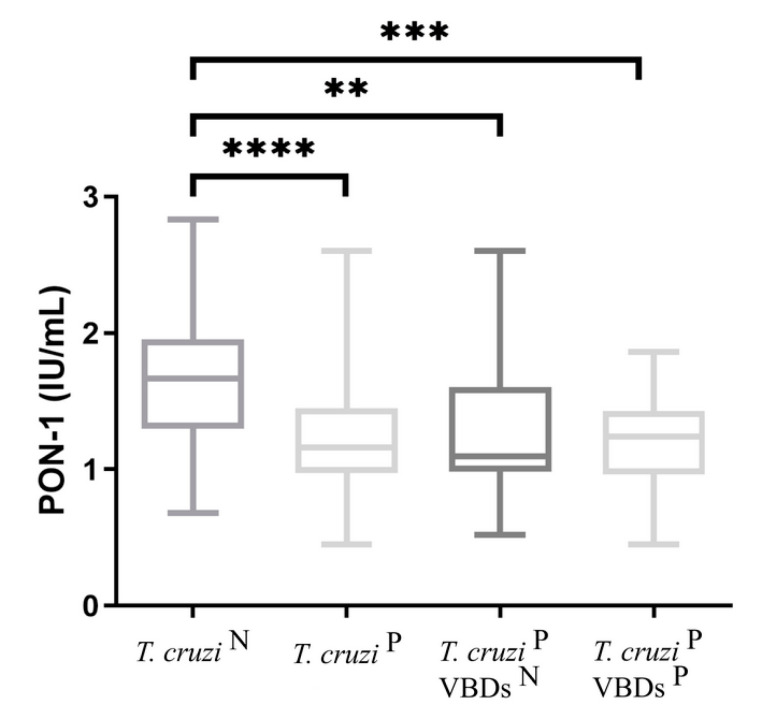
PON-1 activity in the groups: *T. cruzi* ^N^: *Trypanosoma cruzi*-seronegative dogs; *T. cruzi* ^P^: *Trypanosoma cruzi*-seroreactive dogs; *T. cruzi* ^P^ VBDs ^N^: *Trypanosoma cruzi*-seroreactive dogs without seroreactivity to others vector-borne diseases; *T. cruzi* ^P^ VBDs ^P^: *Trypanosoma cruzi*-seroreactive dogs with seroreactivity to others vector-borne diseases. Student’s *t* test was used to compare *T. cruzi* ^N^ vs. *T. cruzi* ^P^. One-way ANOVA followed by uncorrected Fisher LSD test was performed to compare *T. cruzi* ^N^, *T. cruzi* ^P^ VBDs ^N^ and *T. cruzi* ^P^ VBDs ^P^. Asterisks show significant differences between the groups (*p* < 0.05).

The results for analyses of PON-1 obtained in all groups are shown in Figure 2. Median values of PON-1 for group 2 (1.16 IU/mL), 2a (1.095 IU/mL) and 2b (1.24 IU/mL) were significantly lower than those of group 1 (1.665 IU/mL).

### 3.3. Ferritin Concentrations in T. cruzi-Seronegative Dogs (Group 1) vs. T. cruzi-Seroreactive Dogs (Group 2) and T. cruzi-Seroreactive Dogs without (Group 2a) and with Seroreactivity to Others Vector-Borne Diseases (Group 2b)

**Figure 3:**
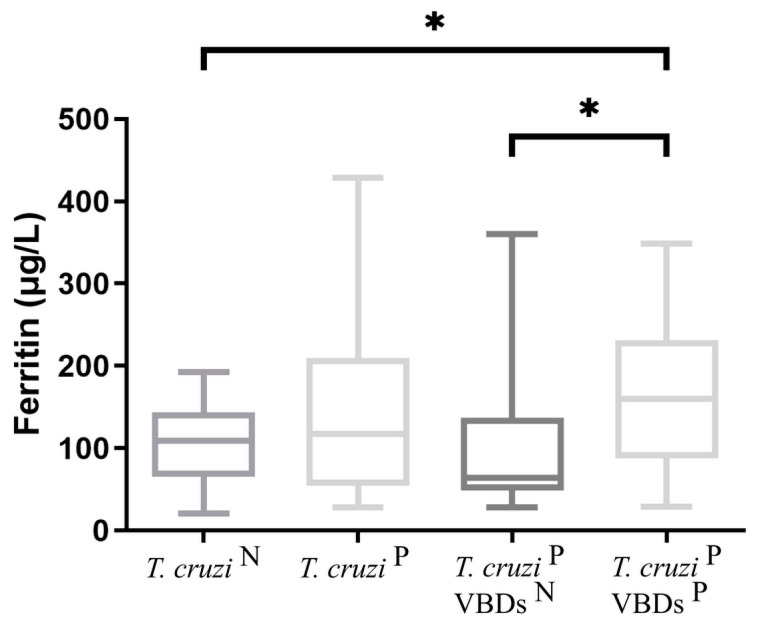
Ferritin concentration in the groups: *T. cruzi* ^N^: *Trypanosoma cruzi*-seronegative dogs; *T. cruzi* ^P^: *Trypanosoma cruzi*-seroreactive dogs; *T. cruzi* ^P^ VBDs ^N^: *Trypanosoma cruzi*-seroreactive dogs without seroreactivity to others vector-borne diseases; *T. cruzi* ^P^ VBDs ^P^: *Trypanosoma cruzi*-seroreactive dogs with seroreactivity to others vector-borne diseases. Student’s *t* test was used to compare *T. cruzi* ^N^ vs. *T. cruzi* ^P^. One-way ANOVA followed by uncorrected Fisher LSD test was performed to compare *T. cruzi* ^N^, *T. cruzi* ^P^ VBDs ^N^ and *T. cruzi* ^P^ VBDs ^P^. Asterisks show significant differences between the groups (*p* < 0.05).

The results for analyses of ferritin obtained in all groups are shown in Figure 3. The median value of ferritin for group 2b (159.9 μg/L) was significantly higher than those of group 1 (109.3 μg/L). Additionally, there was a significant difference when group 2a (64.5 μg/L) was compared with group 2b (159.9 μg/L). No significant differences were found between group 1 and groups 2 and 2a.

### 3.4. CRP and Hp Concentrations in T. cruzi-Seronegative Dogs (Group 1) vs. T. cruzi-Seroreactive Dogs (Group 2) and T. cruzi-Seroreactive Dogs without (Group 2a) and with Seroreactivity to Others Vector-Borne Diseases (Group 2b)

**Figure 4:**
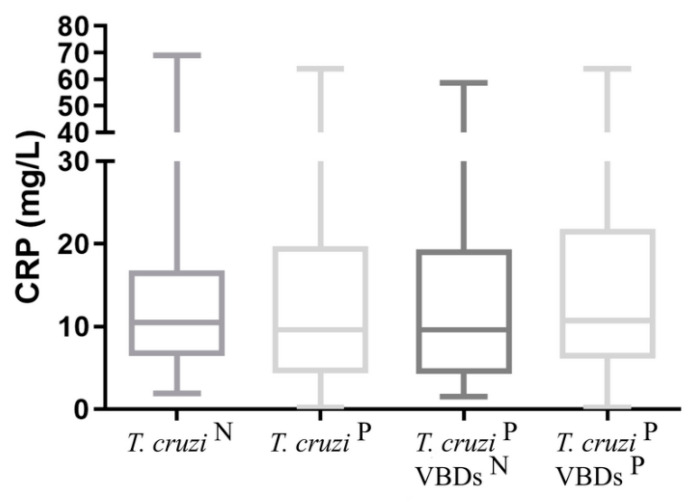
CRP concentration in the groups: *T. cruzi* ^N^: *Trypanosoma cruzi*-seronegative dogs; *T. cruzi* ^P^: *Trypanosoma cruzi*-seroreactive dogs; *T. cruzi* ^P^ VBDs ^N^: *Trypanosoma cruzi*-seroreactive dogs without seroreactivity to others vector-borne diseases; *T. cruzi* ^P^ VBDs ^P^: *Trypanosoma cruzi*-seroreactive dogs with seroreactivity to others vector-borne diseases. Student’s *t* test was used to compare *T. cruzi* ^N^ vs. *T. cruzi* ^P^. One-way ANOVA followed by uncorrected Fisher LSD test was performed to compare *T. cruzi* ^N^, *T. cruzi* ^P^ VBDs ^N^ and *T. cruzi* ^P^ VBDs ^P^. No significant differences between the groups were observed (*p* < 0.05).

**Figure 5:**
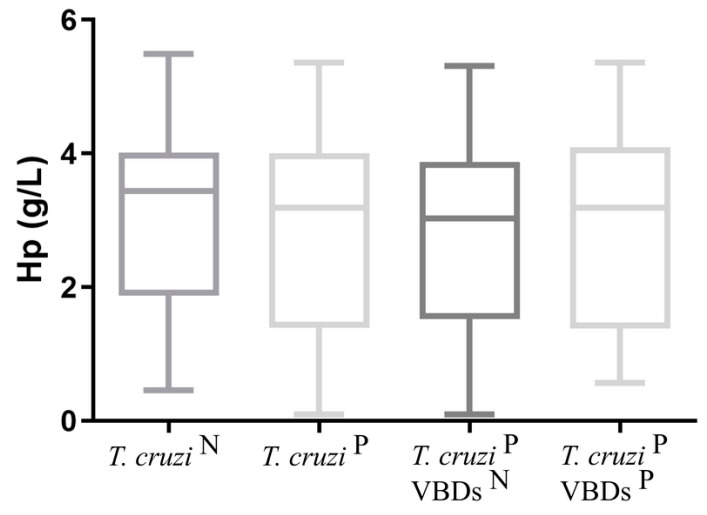
Hp concentration in the groups: *T. cruzi* ^N^: *Trypanosoma cruzi*-seronegative dogs; *T. cruzi* ^P^: *Trypanosoma cruzi*-seroreactive dogs; *T. cruzi* ^P^ VBDs ^N^: *Trypanosoma cruzi*-seroreactive dogs without seroreactivity to others vector-borne diseases; *T. cruzi* ^P^ VBDs ^P^: *Trypanosoma cruzi*-seroreactive dogs with seroreactivity to others vector-borne diseases. Student’s *t* test was used to compare *T. cruzi* ^N^ vs. *T. cruzi* ^P^. One-way ANOVA followed by uncorrected Fisher LSD test was performed to compare *T. cruzi* ^N^, *T. cruzi* ^P^ VBDs ^N^ and *T. cruzi* ^P^ VBDs ^P^. No significant differences between the groups were observed (*p* < 0.05).

The results for analyses of CRP and Hp obtained in all groups are shown in Figure 4 and Figure 5. For CRP and Hp, no significant differences were found between groups 2, 2a, and 2b when compared against group 1.

## 4. Discussion

The presence of anti *T. cruzi* antibodies reported by Rivadeneira, Montes de Oca, Vázquez-Chagoyán, Martínez, Morán, Ochoa, Zambrano, Garg and Varela [42] in dogs from Colón and Calderón, two urban communities of Portoviejo city in the province of Manabí, located on the pacific coast of Ecuador, during 2018, showed the epidemiological relevance of the dog as a sentinel reservoir and risk factor for Chagas disease spread to humans [47]. APPs have been studied in humans and animals to evaluate the therapeutic response and the diagnosis and prognosis of diseases [36]. In humans diagnosed with Chagas disease, APPs have been previously studied [48,49,50]. To the best of the authors’ knowledge, this is the first study that evaluates the changes in selected APPs (CRP, Hp, ferritin and PON-1) in dogs with natural infection with *T. cruzi* with or without seroreactivity to other VBDs.

In this study, a reduction in serum PON-1 was observed in all dogs infected with *T. cruzi*. It has been previously reported that serum PON-1 decreases in inflammatory processes and in some devastating diseases such as parvoviral enteritis, leishmaniosis, and acute pancreatitis [24,51,52]. In this study, the decrease in PON-1 was observed in all dogs infected with *T. cruzi* independently to their seroreactivity to other VBDs. Significant differences found between groups 1 (*T. cruzi* ^N^) and 2a (*T. cruzi* ^P^ VBDs ^N^) could be related to seroreactivity to Chagas disease, to its possible cardiac involvement, and the oxidative processes derived from it [53]. Although it PON-1 serum concentration has been reported to be reduced in lipemic samples and increased in hemolized samples, in the present study lipemic and/or hemolized samples were discarded, and therefore these variables were not considered in the analysis [54].

Even though the serum ferritin values in all groups were within the reference range, the group of *T. cruzi* ^P^ dogs with seroreactivity to other VBDs showed higher values when comparing against the group of *T. cruzi* ^N^ dogs and also against *T. cruzi* ^P^ dogs without seroreactivity to other VBDs, possibly due to the coexistence of several etiological agents. This could indicate that the presence of higher values of serum ferritin in a *T. cruzi* ^P^ dog could suggest the presence of an additional co-infection [35], indicative of seroreactivity both to *T. cruzi* and *Ehrlichia* spp., the most frequent co-infection found in the present study. It has been previously reported that dogs infected with *E. canis*, either natural or experimentally, show an increment in serum ferritin in either acute or chronic phases [55].

In this study, when all the groups of dogs were compared with the group of *T. cruzi* ^N^ dogs, serum CRP and Hp did not show significant differences. These results are different from studies of APP in humans seroreactive to Chagas disease, where increases in serum CRP and Hp were reported [48,49,50]. This might be related to the lack of obvious clinical symptoms of disease in the dogs in our study. However, more research should be done in the future to understand the role clinical symptoms play in the results of APPs clinical laboratory studies.

During the *T. cruzi* chronic silent infection phase of disease, animals seem to reach some sort of equilibrium with the parasite, where most of the myocardium seems to be in a steady state of damage, with no close contact between immune effector cells and endothelial cells or cardiac myocytes [56]; thus, during this phase, there could be no stimulus for the production of acute phase proteins. Further clinical or experimental studies with animals showing evident clinical signs of the Chagas disease should be conducted to check if changes in APPs are observed in those cases.

Most dogs included in the present study did not show clear clinical signs of any VBDs, despite being seroreactive to one or more pathogens. It is difficult to explain why an increment in serum ferritin was found in these animals while the serum levels of Hp or CRP remained low. In other canine diseases such as Leishmaniasis, when increments in serum ferritin were observed, while no significant changes were reported for other APPs it could indicate a situation of subclinical active disease [57] and this should be explored in the future. On the other hand, the lack of expected serum Hp increments could be explained by the existence of subclinical hemolytic or hemorrhagic processes, which are associated with a drop in Hp serological levels, which in turn, could somehow compensate for the Hp serological increments expected during an inflammatory process [16,58,59].

The analysis of the variations in the serum APPs could contribute to a more accurate prognosis for dog patients with Chagas disease as well as provide better information for developing an improved clinical handling of patients. The decrease in serum PON-1 is indicative of the idea that oxidative stress could be occurring in *T. cruzi*-seroreactive patients with an active infection that has not been controlled, and therefore suggesting that an anti-*T. cruzi* treatment should be provided. When elevated serum ferritin concentrations are observed at the same time, it could be an indicator that other VBDs are co-infecting the patient, and therefore serological tests to detect concomitant infections should be conducted and an adequate monitoring and if needed, treatment must be provided.

The limitations of our study included: (a) a reduced sample size, (b) the impossibility of comparing the results of naturally infected dogs with experimentally infected animals due to an animal welfare committee recommendation, (c) the inability of serial sampling of the same animals, (d) the comparison between healthy dogs and those with seroreactivity to *T. cruzi* with each of the diseases studied in this research, (e) not having studied the seroreactive status of other VBDs in *T. cruzi*-seronegative dogs, and (f) not having performed complementary diagnostic tests such as PCR to detect VBD-active infections in animals. When VBDs is referred in the present work, results should be interpreted with caution, because “IDEXX SNAP^®^ 4Dx^®^ Plus” is a rapid test that would need to be confirmed by at least one other diagnostic test based on a different infection detection principle such as PCR or blood microscopical analysis, among others. In the case of the *T. cruzi* serology-based diagnosis used in the present study, the results were more reliable, since two *T. cruzi* ELISA tests (‘Chagas recombinant microELISA’, based on recombinant protein used as plate-sensitizing antigen, and the ‘Chagas microELISA test system’, based on Trypomastigote lysates used as plate-sensitizing antigens, both from Accutrack, Laboratorios Lemos S.R.L. Buenos Aires, Argentina) were used to detect IgG antibodies, and even if they do not discriminate between an active or a past infection, these serology methods are able to detect acute (between 1 and 2 months of onset of infection) and chronic infections [9].

The control of Chagas disease in endemic countries represents a complex action because it must take into account the diagnosis and biochemical alterations in the host, vector control, as well as inherent factors of the ecosystem and socioeconomic activities.

## 5. Conclusions

The findings of the present study suggest that PON-1 levels are reduced in *T. cruzi*-seroreactive animals without evident clinical signs of the disease either being seroreactive or not to other VBDs. In addition ferritin values were increased in *T. cruzi*-seroreactive with seroreactivity to other VBDs. However, no changes were observed in other acute phase proteins such, CRP, and Hp when comparing these same groups of animals. The decrease in PON-1 could suggest the presence of an oxidative stress response but with no evident signs of inflammation in *T. cruzi*-seroreactive animals and further studies should be made to elucidate the reasons for the increase of ferritin in this report.

## Data Availability

No further data is available.
